# Evaluating the Role of Breathing Guidance on Game-Based Interventions for Relaxation Training

**DOI:** 10.3389/fdgth.2021.760268

**Published:** 2021-12-09

**Authors:** Venkata Nitin Chakravarthy Gummidela, Dennis R. da Cunha Silva, Ricardo Gutierrez-Osuna

**Affiliations:** Department of Computer Science and Engineering Texas A&M University, College Station, TX, United States

**Keywords:** stress, deep breathing, biofeedback, casual games, breathing guides

## Abstract

Working in a fast-paced environment can lead to shallow breathing, which can exacerbate stress and anxiety. To address this issue, this study aimed to develop micro-interventions that can promote deep breathing in the presence of stressors. First, we examined two types of breathing guides to help individuals learn deep breathing: providing their breathing rate as a *biofeedback* signal, and providing a *pacing* signal to which they can synchronize their breathing. Second, we examined the extent to which these two breathing guides can be integrated into a casual game, to increase enjoyment and skill transfer. We used a 2 × 2 factorial design, with breathing guide (biofeedback vs. pacing) and gaming (game vs. no game) as independent factors. This led to four experimental groups: biofeedback alone, biofeedback integrated into a game, pacing alone, and pacing integrated into a game. In a first experiment, we evaluated the four experimental treatments in a laboratory setting, where 30 healthy participants completed a stressful task before and after performing one of the four treatments (or a control condition) while wearing a chest strap that measured their breathing rate. Two-way ANOVA of breathing rates, with treatment (5 groups) and time (pre-test, post-test) as independent factors shows a significant effect for time [*F*(4, 50) = 18.49, *p* < 0.001, $\eta_{time}^{2}=0.27$] and treatment [*F*(4, 50) = 2.54, *p* = 0.05, η^2^ = 0.17], but no interaction effects. *Post-hoc* t-tests between pre and post-test breathing rates shows statistical significance for the game with biofeedback group [*t*(5) = 5.94, *p* = 0.001, *d* = 2.68], but not for the other four groups, indicating that only game with biofeedback led to skill transfer at post-test. Further, two-way ANOVA of self-reported enjoyment scores on the four experimental treatments, with breathing guide and game as independent factors, found a main effect for game [$F\left(1,20\right)=24.49,p<0.001,\;\eta_{game}^{2}=0.55$], indicating that the game-based interventions were more enjoyable than the non-game interventions. In a second experiment, conducted in an ambulatory setting, 36 healthy participants practiced one of the four experimental treatments as they saw fit over the course of a day. We found that the game-based interventions were practiced more often than the non-game interventions [*t* (34) = 1.99, *p* = 0.027, *d* = 0.67]. However, we also found that participants in the game-based interventions could only achieve deep breathing 50% of the times, whereas participants in the non-game groups succeeded 85% of the times, which indicated that the former need adequate training time to be effective. Finally, participant feedback indicated that the non-game interventions were better at promoting in-the-moment relaxation, whereas the game-based interventions were more successful at promoting deep breathing during stressful tasks.

## Introduction

Fast-paced workplaces, with their constant interruptions (e.g., emails, text messages, phone calls) and increased time pressure, can lead to frustration and stress (1). While dealing with these myriad tasks, people tend to engage in shallow breathing (2), which can lead to imbalanced levels of oxygen and carbon dioxide within the body (3), further exacerbating stress and anxiety (4). Therefore, learning to avoid shallow breathing in the presence of constant stressors is paramount. A practical solution is to practice deep breathing, a voluntary technique that promotes relaxation by restoring balance to oxygen and carbon dioxide levels and to the autonomic nervous system (5). Toward this aim, in prior work we developed a technique where users learned to perform deep breathing while simultaneously completing a moderately stressful task. In the technique, which we called game biofeedback, the user played a videogame while their breathing rate was measured with a wearable sensor. The videogame was then adapted in a negative-feedback loop, i.e., it became easier if the user's breathing rate was below a target, and more difficult otherwise (6–9). Our prior results showed that this technique helped users transfer the skill of deep breathing to a more difficult or new task.

The current study follows up on this prior work in several key respects. First, we sought to determine whether similar benefits could be achieved using a technique that does not require an external sensor, such as paced breathing. In paced breathing, users are provided with an audiovisual signal that instructs them to breathe in and out at the appropriate times. Second, our prior studies had been conducted in a controlled laboratory setting, so we also sought to evaluate the two types of breathing guides (biofeedback vs. pacing) in an ambulatory setting, as participants carried out their daily activities. This, in turn, would allow us to examine differences in use patterns. Third, we also sought to evaluate a new “endless” game design that addressed limitations of our original game (6–9), in which participants played in short bursts without a strong sense of progression. Finally, we wanted to determine if integrating paced breathing with gaming would be as effective as integrating breathing biofeedback with gaming had been in our prior work. To answer these questions, we considered a between-subject 2 × 2 factorial design with game delivery (game vs. no-game) and type of breathing guide (pacing vs. biofeedback) as independent factors, leading to four experimental interventions: 1- game biofeedback (GBF: our original technique), 2- visual feedback (VBF: displaying the user's breathing rate, without the game), 3- pacing (PACE: playing an audio pacing signal, without the game or biofeedback), and 4- pacing game (GPACE: the game with background audio pacing signal).

We evaluated the interventions through a series of experiments with healthy adult participants. In a first experiment, conducted in a laboratory setting to evaluate the new interventions, we compared the four treatments against a control condition: game-only (GO: playing the game without biofeedback or pacing). In a second experiment with new participants, we compared the four experimental interventions in an ambulatory setting, where participants were asked to practice their assigned intervention whenever they saw fit during the day.

## Background and Related Work

### Stress and Stress Management

Stress is the body's response to external demands or stimuli. Exposure to stressors invokes the “*flight or fight*” response of the sympathetic branch of the autonomic nervous system (ANS). This response is characterized by increased heart rate, breathing rate and pupil dilation, among other physiological responses (10). The parasympathetic branch of the ANS tries to counter this effect, helping reduce the negative effects of stress on the human body and reach homeostasis. This is known as the relaxation response, and is characterized by a reduction in heart rate, breathing rate, and electrodermal activity, and other physiological signals. Though breathing is a simple function of the body, it has a profound effects on a person's physiological state and can be used to attain the relaxation response (11). Shallow breathing (12–24 breaths per minute) initiated from the upper chest (12) leads to an imbalance in blood oxygen levels, affecting the nervous system balance (13), and leads to stress. Deep or diaphragmatic breathing (around 6 breaths per minute), on the other hand, is initiated from the abdominal area, and is shown to increase parasympathetic activity, restore balance, and assist in attaining a calm and relaxed state (13).

A number of methods have been used to help individuals manage stress, such as cognitive behavioral therapy (CBT), yoga, meditation, and biofeedback. CBT is a form of psychotherapy that aims to increase the adaptive coping mechanisms in response to stressors. CBT has been shown to be an effective treatment for stress-related mental disorders, depression, and anxiety (14). But CBT-based methods are time consuming and require trained clinicians, making them costly in terms of both time and money (15), and also resulting in high attrition rates (16). Mind-body relaxation techniques such as yoga and tai-chi have been shown to be effective as self-guided relaxation interventions (17). Mindfulness methods such as meditation, yoga and mindful breathing have been studied as relaxation interventions in healthy individuals, and in people suffering from specific stress-related issues (18, 19). But these methods also suffer from high dropout rates (20) due to the lack of motivation and the unengaging nature of the exercises (21). Further, these techniques are performed in a quiet environment, which may not generalize to real world scenarios (22).

### Deep Breathing for Relaxation and Self-Regulation

Deep breathing has been used in various studies as a relaxation tool (5, 23–25). Inhaling and exhaling affects the oscillations of the heart rate through a process known as respiratory sinus arrhythmia (RSA). The beat-to-beat (R-R) interval is shortened during inhalation and is prolonged during exhalation (26, 27). High amplitude of heart rate oscillations have been associated with higher relaxed states when an individual performs deep breathing at the resonant frequency of the cardiovascular system, which is close to 0.1 Hz (6 breaths/min) (28).

Various tools and protocols that assist the user in performing deep breathing by means of a pacing signal have been developed to help users relax and self-regulate. Pacing signals are simple breathing aids that dictate a breathing pattern using audio or visual cues. Deep Breaths is a mobile tool that provides the user with a stationary pacing signal in order to attain a relaxed state (29). Paced Breathing is a commercial application available on Google Play Store, where the user can set different breathing patterns that allows the user to achieve resonance breathing rate that maximizes relaxation. Cheng et al. (24) showed that experimental groups using visual guided deep breathing obtained larger standard deviation of beat-to-beat intervals and normalized low frequency power of heart rate variability (HRV), which are indications of a relaxed state, as compared to a control group with no mindful breathing (30). Moraveji et al. (31) studied the influence of peripherally integrated respiration pacing methods into the user's desktop screen, and found that the participants' respiration rate decreased significantly when the screen brightness updated according to a pacing signal.

Various studies have shown the effectiveness of biofeedback games in helping users relax and also learn self-regulation in the presence of stressors (32–35). Sonne and Jenson (34) used a breath-controlled game, ChillFish, to treat children with attention-deficit hyperactivity disorder (ADHD). In ChillFish, children controlled the size of a puffer fish by controlling their breathing. Breathing slowly made the puffer fish bigger, allowing them to collect more rewards. The authors reported a significant increase in HRV, when compared to activities like talking and playing Pacman. Lobel et al. (36) developed a horror-themed biofeedback game called Nevermind to improve the player's emotion regulation skills in the face of a stressful situation. The authors designed the game such that negative arousal amplifies the game's horror setting using heart-rate biofeedback. The authors reported anecdotal evidence collected from three participants, but a comprehensive analysis of the effects of game play was not presented. Osman et al. (32) implemented ubiquitous biofeedback to track mental stress using HRV in a game named Botanical Nerves. In this game, the health of a tree was dependent on the status of the player's ANS. Hence, the tree wilted when the player was feeling stressed, and grew greener when the player was relaxed. In ubiquitous biofeedback, biological monitoring is not time bound to the duration during which the application is used. The author's short-term experiments showed that the game scores and tree health were correlated with the user's relaxation. Their extended experiments, carried over a period of 5 days, showed that participants assigned to the biofeedback group had a healthier tree compared to the non-biofeedback group, indicating the participants were relaxed for longer duration during the 5 days.

Frey et al. (37) designed a biofeedback pendant that could estimate the participant's breathing rate and send biofeedback in real-time via visual, audio, or haptic feedback. The authors investigated whether sharing one's breathing rhythm with a companion could be used to promote bonding and breathing syncing. They observed that participants modified their breathing patterns to mimic the biofeedback with the goal of understanding the underlying emotion experienced by their companion. Participants reported being inclined to sharing their breathing rate with a relative or life partner to relate to their emotion during the day or use it in emergency situations.

Shih (38) developed an approach to detect breathing rate using the smartphone's microphone and used it in a breathing biofeedback game. The objective of their game was for the user to speed-up a sailing boat following a slow-breathing pattern. As the sailboat moved, the landscape quickly changed to provide additional motivation. The biofeedback affected the wind strength, and acceleration and speed of the sailboat—the better the paced breathing was followed, the faster the boat sailed. The authors conducted a within-subject study to compare their biofeedback game against a paced-breathing application without biofeedback. They found that their biofeedback game was significantly rated as more enjoyable, but also more difficult to use than the paced-breathing application. Further, the biofeedback game led to a higher high-frequency HRV component than the paced-breathing application.

In a recent study, Brammer et al. (39) conducted breathing biofeedback training for police officers with the aim of reducing the short-term and long-term negative impact of stress on performance and mental health. In their study, police officers played a virtual reality (VR) game where they were placed in a poorly lit garage surrounded by zombies that could be either benign or hostile depending on their eye-color and body shape. The goal of the game was to shoot the hostile zombies and save the benign ones. The game delivered an intuitive biofeedback representation by modifying the trainee's peripheral vision and environmental lights. Upon lowering their breathing rates, the trainee's peripheral vision widened, and lights became brighter to enable a better in-game performance. Participants played the game with and without biofeedback in multiple alternated sessions, and the authors observed that mean breathing rate decreased over sessions but was significantly lower during the biofeedback sessions.

### Biofeedback Games for Skill Transfer

Skill transfer is the ability to effectively transfer the skills learned with assistance on a task to a different task with no assistance. A few studies have investigated whether relaxation skills learned with biofeedback games could be transferred to another task, performed without biofeedback (40–42). Larkin et al. (41) studied skill transfer using heart-rate feedback and contingent reinforcement. In their study, participants were shown their heart rate as a peripheral visual cue and a score contingency was imposed to improve the reinforcement of the participant's ability to maintain their heart rate lower. Participants were assigned to one of the three experimental groups: score contingency (SC), visual feedback (FB), and a combined strategy (SC-FB). They showed that SC-FB and SC groups had significantly lower heart rate both during game play without feedback and while performing a novel mental arithmetic task. Bouchard et al. (43) studied whether a stress-management technique known as immersion and practice of arousal control training (ImPACT) was better than ‘training as usual' when delivered to military personnel. In ImPACT, soldiers were exposed to a stressful situation through immersion in a horror/first person shooter game, where they try to learn skills with the use of biofeedback. Their study, with 60 participants, showed that the ImPACT group obtained better self-regulation skills compared to the control group, measured through salivary cortisol and heart rate. The ImPACT group also obtained better task performance than the control group. Lewis et al. (44) studied the impact of heart rate variability biofeedback in stress relaxation training to counter Post Traumatic Stress Syndrome (PTSD) and depression symptoms. They used a relaxation training protocol known as pre-deployment stress inoculation training (PRESTINT), which is a slow paced-breathing training supported by HRV biofeedback in a simulated combat-training exercise. The authors showed that the participants were more relaxed as observed using HRV in a post-training combat simulation designed to heighten arousal.

Parnandi et al. (33) developed a respiratory biofeedback game named Chill-Out that penalizes fast and shallow breathing by increasing the game difficulty to enforce deep breathing skills. An experimental study with the biofeedback game showed that participants were able to control their breathing in a subsequent stressful task, as compared to a control condition. In later studies, Parnandi and Gutierrez-Osuna (6, 7) studied various biofeedback strategies to increase the skill acquisition and transfer to a subsequent stressful task. They compared various game biofeedback strategies, such as visual feedback, game adaptation, and combined biofeedback, to study which would promote better relaxation, skill acquisition, and skill transfer. The authors concluded that using a combined strategy of visual feedback and game adaptation provided higher skill retention than the other conditions. They concluded that game adaptation allowed participants to reduce their breathing rate whereas visual feedback helped maintain it at the desired breathing rate range. The authors also observed that the combined strategy led to the fastest acquisition of deep breathing skills.

Blum et al. (45) conducted a study comparing VR and standard (non-VR) HRV biofeedback in terms of skill transfer, self-regulation, focus, and attentional resources. Participants in the standard HRV biofeedback condition received abstract feedback by means of graphical geometrical indicators, while an audio pacing signal played along with ambient instrumental music. Participants assigned to the VR HRV biofeedback were presented with a virtual beach scenery at sunset, with the feedback parameter bound to the cloud coverage—the better the performance, the clearer the sky. Participants assigned to both groups completed the Stroop Color-Word Task (CWT) before and after treatment, which was used for pre- vs. post-test analysis. The authors found the VR HRV biofeedback condition outperformed the standard HRV biofeedback condition in terms of relaxation, relaxation self-efficacy, promoting better focus and less mind-wandering, attentional resources, but not in terms of HRV.

## Methods

As indicated in the previous section, deep breathing exercises and the combination of biofeedback and videogames can be very successful at inducing relaxation and promoting skill transfer. In this study, we sought to examine the integration of a casual game with two distinct types of breathing guides as relaxation aids. The first guide is biofeedback, which provides users with information about their breathing rate, either directly (i.e., a numeric display) or indirectly (i.e., via game adaptation). The second guide is by means of an audio pacing signal that dictates a breathing rhythm that tends to promote relaxation.

For this purpose, we designed four experimental treatments following a 2 × 2 factorial design with game delivery (game vs. no-game) and type of breathing guide (pacing vs. biofeedback) as independent factors. Summarized in Table 1, these were: game biofeedback [GBF: our original technique (6–9)]; visual biofeedback (VBF: displaying the user's breathing rate, without the game); pacing (PACE: playing an audio pacing signal, without the game or biofeedback); and pacing game (GPACE: the game with background audio pacing signal). Before we describe these four treatments, we introduce the casual game that formed the basis of the two game-based interventions. Then, we describe the two stressor tasks that were used to assess skill transfer.

**Table 1:** Four interventions resulting from a factorial design with game delivery (game vs. no-game) and type of breathing guide (pacing vs. biofeedback) as independent factors.

		**Breathing guidance**	
		**Biofeedback**	**Audio pacing**
Game	Yes	Game Biofeedback (GBF)	Game with Pacing (GPACE)
	No	Visual Biofeedback (VBF)	Pacing (PACE)

### Base Casual Game

**Figure 1:**
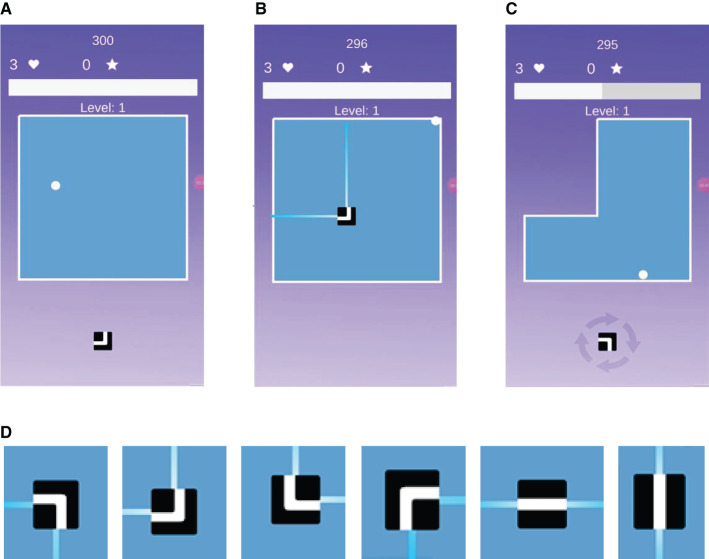
Screen shots of the casual game (scale). **(A)** A square arena is initially presented to the user. **(B)** Upon placing the tile in the arena. **(C)** The arena gets cropped. **(D)** Illustration of the six tiles available in the game, and their respective cropping actions.

As the base game, we developed a clone of the casual puzzle-type mobile game Scale. This is considered and “endless” game^1^, since it can be played indefinitely, a characteristic that we thought would be important to promote sustained practice when integrated with a breathing guide. As illustrated in Figures 1A–C, the player is initially presented with a square arena, in which a ball is bouncing with a randomly initialized direction and location. The objective of the game is to crop the arena using the tile shown at the bottom of the screen. Six different tiles are available (see Figure 1D), and each one cuts the arena based on its shape and orientation by projecting rays toward the ends. The default speed of the ball and the projection lines are set such that it takes either 1 s to cross a full-width arena. If the ball hits the rays while they are expanding (i.e., before they reach the bounds of the arena), the player loses a life. Therefore, the player must control the timing and placement of the tiles to maximize the area that is cropped while avoiding hitting the ball. Once 50% of the area is cropped, the player advances to the next level, and the screen zooms in to fit the remaining arena. This process repeats indefinitely, from which it derives its “endless” nature. The player's score grows according to the sum of all levels completed, e.g., after finishing level 3, the player has 6 points: one point for level 1, two points for level 2, and three points for level 3. In each game session, the player has three lives. If players lose all the lives, they are given the option to play again. The new game starts at level 1, but the total score from the previous game play is carried over to encourage the player to improve their best score. The game was developed using the Unity game engine.

### Biofeedback-Based Interventions

We consider two biofeedback interventions to deliver information to the user about their breathing rate. The first intervention, game biofeedback, delivers this information primarily in an indirect fashion, by altering how the game plays. The second intervention, visual biofeedback, delivers it directly, via a numeric indicator.

#### Treatment 1: Game Biofeedback

The game biofeedback (GBF) treatment uses instrumental conditioning as its core mechanism on top of the base game described in section Base casual game. In instrumental conditioning, the user is presented with rewards or penalties based on their response. In particular, GBF uses negative reinforcement instrumental conditioning (NR-IC^2^), where the target behavior (staying calm and relaxed) eliminates the occurrence of an averse stimulus (increased game difficulty). Under the NR-IC system, the player must try to control their arousal (breathing rate) so as to keep the game difficulty lower and progress in the game and score higher. NR-IC has been shown to increase the likelihood of the target behavior to be repeated in the future (47).

**Figure 2:**
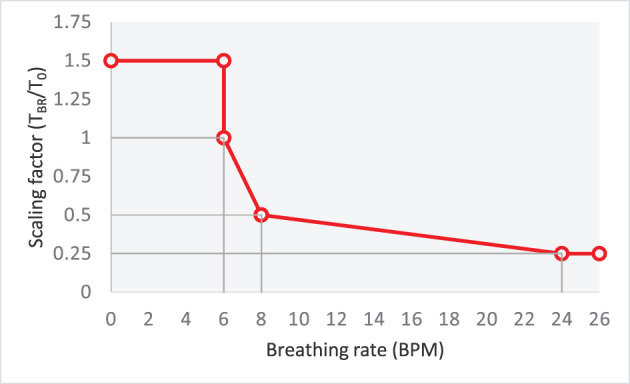
Relationship between the scaling factor $\left(\alpha =\frac{T_{BR}}{T_{0}}\right)$ and breathing rate.

Biofeedback is delivered primarily^3^ via game adaptation, by altering the difficulty of the game in response to the player's breathing rate. In particular, we control the difficulty of the game with two mechanisms. First, we change the speed at which the ball travels, by adapting the time it takes for the ball to travel one full-width arena. The scaling factor [α : the ratio of the time taken for the ball to travel the arena at a breathing rate BR (*T*_*BR*_) and the default travel time (*T*_0_)] follows a piecewise linear function with respect to the breathing rate; see Figure 2. For breathing rates <6 bpm, the travel time of the ball (*T*_*BR*_) is 1.5*T*_0_ but drops to *T*_0_ just above 6 bpm. At 8 bpm, the travel time is 0.50 × *T*_0_, and at 24 bpm it is 0.25 × *T*_0_. As a second game-adaptation mechanism, we add a new ball to the arena whenever the player's breathing rate crosses the target of 6 bpm. This serves the function of alerting the player that their breathing rate has exceeded the target. The second ball remains in the arena for as long as the player's breathing rate is above 6 bpm *and* increasing. However, as soon as the breathing rate starts to decrease (even if it is still above 6 bpm), one of the two balls in the arena is randomly removed; this gives the player an opportunity to quickly recover. Table 2 summarizes the game adaptation with respect to the breathing rate and its rate of change.

**Table 2:** Effect of breathing rate and its rate of change on the number of balls in the arena (*N*_*b*_) and ball travel time *T*_*BR*_. Scaling factor (**α**) for BR > 6 is obtained from Figure 2.

	***BR* ≤ 6**	**BR>6**
Δ*BR* < 0	*N*_*b*_ = 1 *T*_*BR*_ = 1.5*T*_0_	*N*_*b*_ = 1 *T*_*BR*_ = α*T*_0_
Δ*BR*≥ 0	*N*_*b*_ = 1 *T*_*BR*_ = 1.5*T*_0_	*N*_*b*_ = 2 *T*_*BR*_ = α*T*_0_

#### Treatment 2: Visual Biofeedback

**Figure 3:**
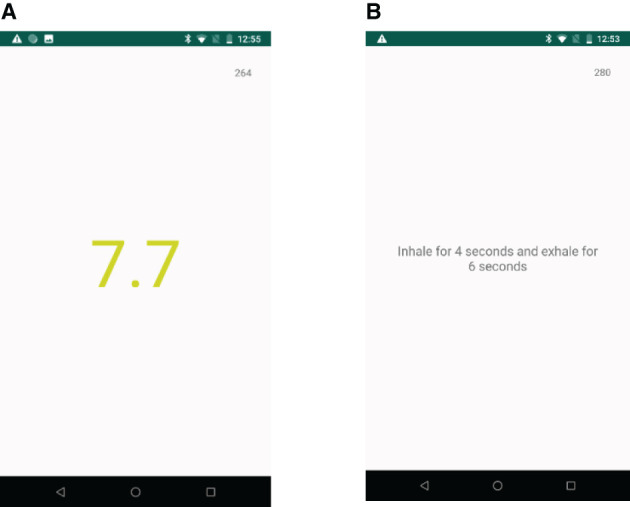
Screen shot of the visual feedback application **(A)**, and the pacing application **(B)**.

The visual biofeedback (VBF) intervention is a straightforward application of biofeedback, where the user is provided with their physiological information (breathing rate in this case). We deliver this in the form of a visual cue using a numerical indicator of the player's breathing rate; see Figure 3A. To make the biofeedback more obvious, the number's font color changes to green when the breathing rate (BR) is in the desirable range (*BR* ≤ 6 *bpm*), yellow while approaching the desirable range (6*bpm* < *BR* < 12 *bpm*), and red when the breathing rate is far from the desirable range (*BR* > 12*bpm*).

### Pacing-Based Interventions

Pacing signals are breathing aids that dictate a pattern for the participant to follow, e.g., when to inhale and when to exhale. These signals are often delivered in a visual or auditory fashion. In our study, we use an audio pacing signal delivered by increasing the intensity of a relaxing sound during inhalation and reducing it during exhalation. The inhalation duration is set to 4 s and exhalation to 6 s (6 bpm). We use this frequency as prior works has shown that the cardiovascular system has a resonant frequency of 0.1 Hz (6 bpm) (28), and shorter inhalation and longer exhalation lead to higher respiratory sinus arrhythmia (26) causing higher relaxation. In the following sections, we describe the two pacing-based interventions: Game with Pacing (GPACE) and Pacing (PACE).

#### Treatment 3: Game With Pacing

We implemented this adaptation to test the effect of using guided deep breathing in the presence of a primary task (the game). We provide the same pacing signal (4 s for inhale and 6 s for exhale) as a background audio during with the game. The speed of the ball remains unchanged during game play, and only one ball is present in the arena. Moraveji et al. (31) showed that participant's respiration rate decreased significantly by providing a respiration pacing guide in a visual peripheral manner while performing a primary information work task. Thus, this intervention can be thought of as providing the respiration guide peripherally, in the form of audio cues while the primary task is the game.

#### Treatment 4: Pacing

For this intervention, we developed a simple application that plays the auditory pacing signal that increases in intensity for 4 s (inhale) and decreases in intensity for 6 s (exhale) till it reaches the original intensity level. The audio pacing signal is played, with a text in the center that says, “Inhale for 4 seconds and exhale for 6 seconds”; see Figure 3B.

### Stressors

**Figure 4:**
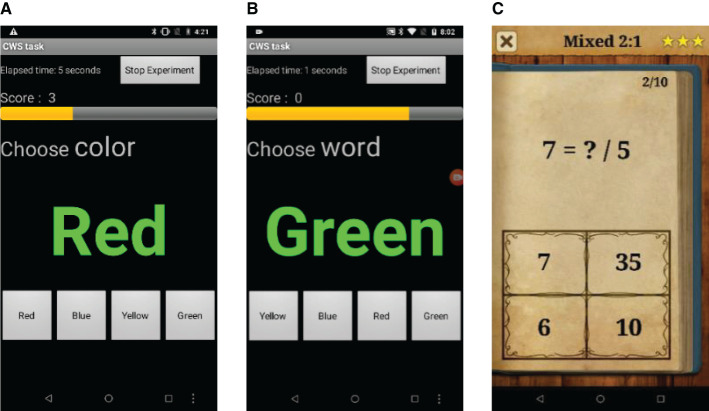
Color word test (CWT) operating in **(A)** incongruent mode and **(B)** congruent mode. **(C)** King of math (KOM) mental arithmetic task in mixed setting.

Following prior work (7), we use two assessment tasks to examine whether the interventions lead to skill transfer: the Stroop color word test (CWT) (48), and a mental arithmetic task. The Stroop CWT is widely used to induce arousal through cognitive work load. We used a modified version of the CWT. In the conventional CWT, four color words (red, blue, green, and yellow) are shown in a different ink (e.g., red) and the participant is asked to choose either the displayed word or the ink color displayed. Our method switches between congruent and incongruent modes every 30 s. In congruent mode, the word and the ink color are the same, whereas in incongruent the word and ink color differ; see Figures 4A,B. Further, the location of the answer buttons is also randomized every time. Every correct answer increases the score by one while each wrong answer decreases it by one.

Following our prior work (7), we also use a second assessment task: mental arithmetic via the game King of Math (KOM), available on the Google Play store. In this application, the player tries to score as high as they can by answering basic arithmetic tasks such as additions, multiplication, and fractions in a limited time. As the level increases, the level of difficulty of the arithmetic increases. The participants are presented with four answer choices—see Figure 4C. We use a mixed setting in the game, which shows an assortment of questions based on various math concepts. Each level has 10 questions, and the score is based on the number of questions correctly answered by the participant. Additionally, if the participant commits three mistakes, they are not allowed to progress and the level restarts.

## Experiment 1: Laboratory Study

In a first experiment, we evaluated the four breathing interventions in a laboratory setting. The study was designed in a between-subjects fashion to minimize fatigue (due to performing all the tasks), carryover (due to first treatment interfering in the second), and learning effects (better performance in the assessment tasks due to unexplained factors of the treatment interference). Participants were randomly assigned to one of the four interventions (VBF, PACE, GBF, or GPACE) or to a control group (Game only, or GO).

We recruited 30 healthy participants (16 males, 14 females; average age: 23 years; std.: 4.25 years) using the Texas A&M University bulk mail service. Participants were all university staff and students and had to meet three inclusion criteria: age between 18 and 35 years, no self-reported history of clinical anxiety or depression, and be a fluent speaker of English. Participants had experience using mobile phones and playing causal mobile games but none reported experience with biofeedback applications. The Texas A&M University Institutional Review Board^4^ approved our experiments prior to conducting the studies. Written informed consent was obtained from participants before starting the experiments. Participants were asked to place a Zephyr Bioharness chest strap snugly around their sternum (which was used to measure their breathing rate) and were asked to assume a comfortable seating position.

The study consisted of five stages:

- Pre-Test: Participants performed the CWT for 3 min. Participants had 3 s to answer each question. This task was performed to measure participants' arousal under stress without training.
- Paced Breathing: Participants followed an audio breathing guide set to a 4-s inhale and a 6-s exhale. This task was performed for 3 min to familiarize participants with the target breathing rate (6 bpm).
- Training: Participants played an unaltered version of the casual game (section Base casual game) for 3 min, to familiarize themselves with the game mechanics.
- Treatment: Participants were assigned to one of five groups (GBF, GPACE, VBF, PACE, GO) and they performed the corresponding treatment for 6 sessions, each session lasting 5 min, with a 30–60 s break between sessions. Participants assigned to game-based groups were informed of their game score between sessions and were asked to improve it.
- Post-Test: Participants performed the CWT again for 3 min, but the difficulty was increased (2 s to answer instead of 3 s) to account for learning effects.

To ensure a fair comparison between the game groups, we needed to keep the difficulty level of the games similar across all game groups. The GBF group has dynamic control over the game difficulty based on breathing rate, whereas the GO and GPACE groups did not experience a change in game difficulty. To make the game equally challenging for all three conditions, we employed a yoked design, where we first conducted the experiments for the GBF group and recorded the ball speed during treatment. We then set the ball speed for the GO and the GPACE groups as the average speed during GBF.

### Results

#### Breathing Rate

**Figure 5:**
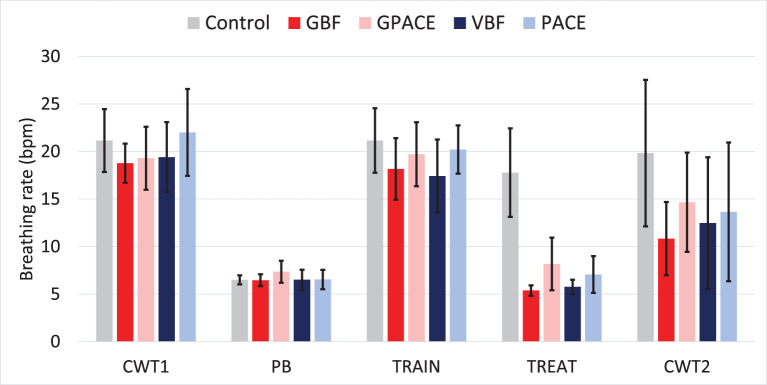
Average breathing rate per group during Pre-Test (CWT1), paced breathing (PB), Training (TRAIN), treatment (TREAT), and Post-Test (CWT2).

Figure 5 shows the average breathing rates for all groups at the different stages of the protocol. The five groups had similar breathing rates during the CWT1, PB and TRAIN stages (one-way ANOVAs showed no statistically-significant differences). We observed an average of 20 bpm across all groups during Pre-Test, which is expected due to the mild stressor delivered by the CWT1. We also observed breathing rates around 6 bpm during PB, which shows that participants were able to follow the pacing signal successfully.

During the treatment phase, the four experimental conditions were able to lower participants' breathing rates significantly when compared to the control group, as indicated by one-way ANOVA: *F*(4, 25) = 21.08, *p* < 0.001, η^2^ = 0.77. *Post-hoc* pairwise two-sample *t*-test comparisons using Bonferroni adjusted alpha levels of 0.005 per test (0.05/10), confirm that there are statistically significant differences between the control group and each of the four experimental treatments (*p* < 0.001) but no differences between pairs of experimental groups. This indicates that adding a gaming component to the breathing guide (pacing or biofeedback) did not alter the breathing rates during treatment. Finally, we performed two-way ANOVA on the four experimental treatments, with breathing guide and game as independent factors. We observed no significant effect for game and no interactions, but we found a main effect for breathing guide [$F\left(1,20\right)=8.39,\;p=0.008,\;\eta_{guide}^{2}=0.30$], indicating that the average breathing rate for the biofeedback groups (5.57) was lower than that for the non-biofeedback groups (7.90).

Then, we compared the average breathing rate during the CWT1 and CWT2 phases to determine which group was able to control their breathing rate the most. As shown in Figure 5, the average breathing rate for the control group remained relatively stable from CWT1 to CWT2, whereas all experimental treatment groups had lower breathing rates during CWT2 than during CWT1. To examine whether these differences were significant, we performed 2-way ANOVA with treatment (5 groups) and time (CWT1 vs CWT2) as independent factors. We observe a significant effect for time [$F\left(4,50\right)=18.49,p<0.001,\;\eta_{time}^{2}=0.27$] and treatment [$F\left(4,50\right)=2.54,p=0.05,\;\eta_{time}^{2}=0.17$, but no interaction effects. To examine to which treatment these differences could be attributed, we performed one-sample *t*-tests between breathing rates at CWT1 and CWT2 for the five groups using Bonferroni adjusted alpha levels of 0.01 per test (0.05/5). This analysis shows statistical significance for the GBF group [*t*(5) = 5.94, *p* = 0.001, *d* = 2.68], but not for the other four groups (*p*>0.01). Thus, while all the experimental treatments led to a reduction in breathing rate from CWT1 to CWT2, implying some level of skill transfer, this reduction was only significant for GBF.

**Figure 6:**
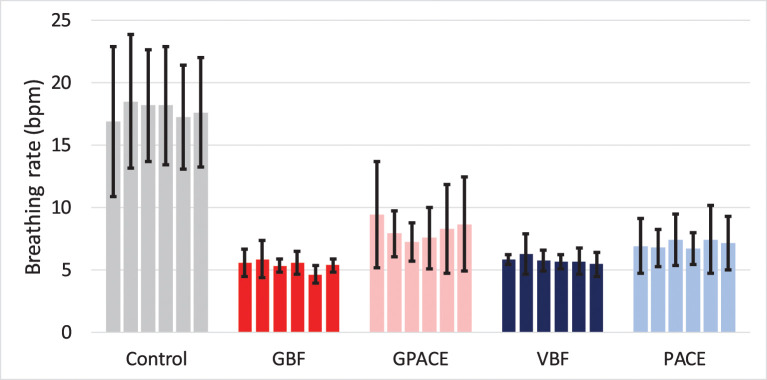
Average breathing rate trend during the six-treatment session for control and experimental groups.

Figure 6 shows the breathing rate during the six treatment sessions for all groups. One-way ANOVA for each of the five treatments show that the effect of time was not significant, which indicates that participants learned to perform the exercise within the first session. The control group has an average breathing rate close to 19 bpm with a very high variance, followed by GPACE with an average breathing rate close to 8 bpm, then PACE around 7 bpm. Finally, VBF and the GBF groups were able to aid the participants in controlling their breathing rate close to 6 bpm.

#### Survey and Subjective Analysis

**Figure 7:**
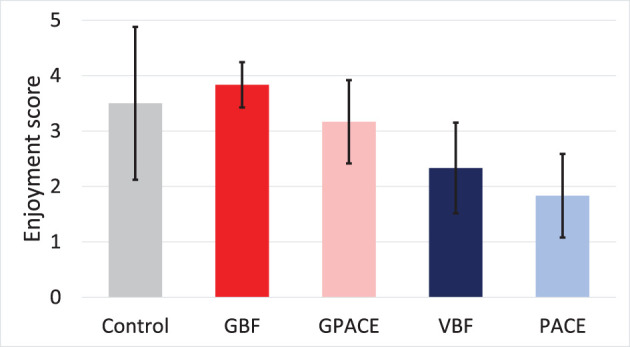
Enjoyment score reported by the participants across the five treatments.

At the conclusion of the experiment, participants were asked to fill a survey regarding their experience with the treatments. We asked how enjoyable the participant found the treatment on a scale of 1–5. Figure 7 shows the perceived enjoyment for all the groups in the study. One-way ANOVA shows that the effect of treatment was statistically significant: *F*(4, 25) = 5.34, *p* = 0.002, η^2^ = 0.46. Further, we performed two-way ANOVA on the four experimental treatments, with breathing guide and game as independent factors. We observed no significant effect for breathing guide and no interactions, but we found a main effect for game [$F\left(1,20\right)=24.49,\;p<0.001,\eta_{game}^{2}=0.55$], indicating that the average enjoyment for the game groups (3.50) was higher than that for the non-game groups (2.08). These results suggest that games may be used to increase the engagement of relaxation interventions, and therefore counteract the dropouts rates that are observed in other interventions (20, 21).

Finally, we examined subjective assessments provided by the participants. Participants in the VBF groups found it easy to control their breathing rate, as mentioned by the comments “*It was easy to control breathing because the screen showed your breathing rate*.” A participant in the PACE groups mentioned “*It's too monotonic and I got bored after the second trial*” indicating that a pacing signal would lead to boredom and distraction. Participants in the GBF found the biofeedback integration helpful in controlling their breathing rate as mentioned by the comments, “*I like the idea that you can control the game with your breathing and score more points*,” and another participant mentioned “*The game play was fun and the pop-ups and the breathing rate indicator were helpful*.” Further, participants in the GPACE groups found the pacing signal distracting from the game play, as one participant mentioned “*I kept switching back and forth between playing the game and breathing*” whereas another participant noted that “*After 3-4 trials I just felt like only playing the game or breathing*” indicating that the pacing signal, though integrated into the game, was perceived as an additional task. When participants from the GBF and GPACE groups were asked if they were able to concentrate on the game, all 6 participants from GBF answered yes, compared to only 3 participants from GPACE. This is an indication that the game and the biofeedback mechanism are acting together to help the participant perform both tasks better, whereas the pacing signal works against game play and acts as a distraction. Finally, a participant in the control group mentioned “*I was more relaxed during paced breathing and the game didn't really help me relax*,” which suggests that the game by itself does not provide any means of relaxation.

## Experiment 2: Ambulatory Study

Relaxation interventions are generally studied when the treatment is performed in a controlled setting, which makes it likely that participants pay close attention to the treatment. However, without the supervision of study staff, the user may not perform the treatment in the intended way and as a result may not draw the desired benefits. Further, some treatment interventions may appeal to users better than others. This is a very important feature of mobile interventions, as they are generally self-administered. To address these questions, in Experiment 2 we conducted an ambulatory study with users practicing the four experimental interventions^5^ in free-living conditions. A total of 36 healthy participants (15 male, 21 female; average age: 23 years; std.: 4.25 years) were recruited following the same procedures as those described in Experiment 1. The primary objectives of Experiment 2 were to analyze usage statistics of the various treatments and their ability to help participants control their breathing rate in an ambulatory setting. We also examined whether the training led to skill transfer, and participants' subjective experiences.

### Protocol

We divided the ambulatory study into three stages, Pre-Treatment, Treatment, and Post-Treatment, as described below. In the Pre-Treatment stage, participants performed four tasks:

- Baseline: Participants watched a short relaxing video for 2 min^6^.
- Pre-Test: Participants performed the CWT for 5 min, with a 3-s response time.
- Paced Breathing: Same as in the laboratory study in Experiment 1.
- Training: Participants were randomly assigned to one of four groups (GBF, GPACE, VBF, PACE), and they performed the corresponding treatment to learn the mechanics of the treatment intervention for 5 min.

Treatment stage: Once the training task was finished, participants left the lab and went about their day as usual, but were asked to perform the assigned treatment intervention (5 min) whenever they saw fit during the day. Participants were given a Nexus 6P smartphone with the corresponding application installed, and were asked to continue wearing the Zephyr Bioharness chest strap. Participants in the pacing groups also received disposable headphones so the sound would not inconvenience people around them. This treatment stage starts once the participant stepped out of the lab and ends once the participant returned to the lab at the end of the workday (~7 h).

Upon return to the laboratory at the end of the day (Post-Treatment stage), participants performed an additional three tasks.

- Baseline: Same as the initial baseline session.
- Treatment: Participant performs their assigned treatment task for a final time.
- Post Test: Participant performs the CWT again, and also the unseen mental arithmetic task (KOM), each for 3 min. As in the laboratory study, the response time in CWT was reduced to 2 s.

### Results

#### Adherence to Treatment

**Figure 8:**
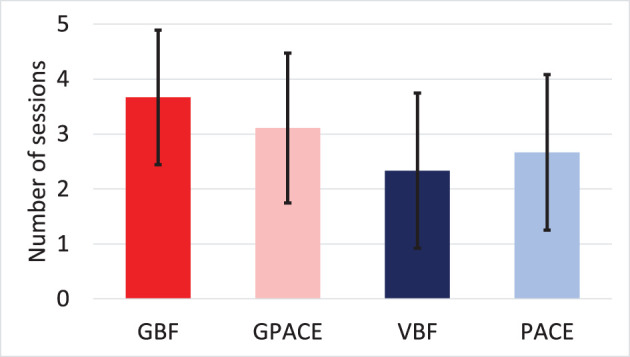
Average number of times the treatment intervention was used during the treatment stage.

In a first analysis, we examined participants' adherence to their assigned treatment. Figure 8 shows the average number of times participants used the treatment throughout the 7-h treatment stage. To analyze these results, we performed 2-way ANOVA with breathing guide and game as independent factors. We observed no significant effect for breathing guide and no interactions, but a marginally significant effect for game [$F\left(1,32\right)=3.86,\;p=0.058,\eta_{game}^{2}=0.11$]. To further examine this result, we performed a two-sample *t*-test by combining participants in the two game interventions into one group (GBF, GPACE) and participants in the two non-game interventions into a second group (VBF, PACE). We observed a statistically significant difference between the two groups [*t*(34) = 1.99, *p* = 0.027, *d* = 0.67], which suggests that the addition of a game component improved adherence.

#### Treatment Effectiveness

**Figure 9:**
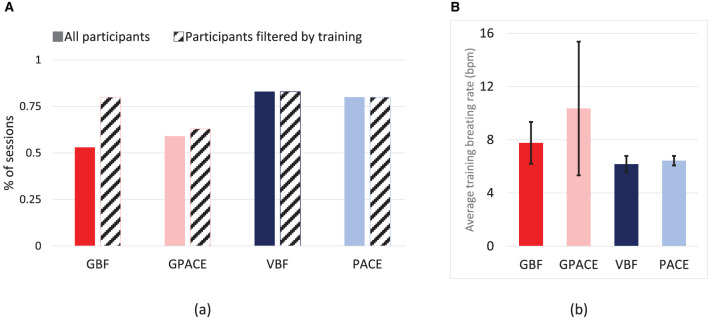
**(A)** Percentage of treatment sessions where the breathing rate of the last 30 s was in the effective breathing range (6BPM ± 0.9BPM) for participants, and only the participants with average training breathing rate in the effective dosage range. **(B)** Average breathing rate (BPM) during training session for all groups.

In a controlled setting there is a high chance that the treatment will be performed in the desired manner. However, when left to the participant's discretion to use the application, several factors such as the participant's interest level, amount of time they can spend on the task, and their physiological state may affect the way the treatment is used. For this reason, we assessed the effectiveness of the various treatments in helping participants maintain a low breathing rate by analyzing the last 30 s of the treatment sessions. The solid bars in Figure 9A show the percentage of treatment sessions where participants were able to maintain their breathing within the range 6 *bpm* ± 0.9 *bpm*, which we define as the effective breathing range^7^. As shown, both non-game interventions (VBF, PACE) were successful in ~85% of the trials, whereas the game interventions had a much lower success rate, around 50%. A likely explanation for this result is that VBF and PACE are simple tasks, whereas GBF and GPACE require multi-tasking, and therefore require more practice to internalize both the game and the breathing maneuver. To corroborate this explanation, Figure 9B shows the average breathing rate during the training session at the start of the study day. As shown, the average breathing rate for participants in the GBF and GPACE groups was significantly higher than the effective breathing range, which suggests that participants in those groups did not receive sufficient training^8^. Accordingly, we examined the percentage of treatment sessions where participants reached the effective breathing range in the last 30 s, when only considering participants whose average breathing rate during the training session had been in the effective breathing range. Results are shown by the diagonally-striped bars in Figure 9A. We noticed that the percentage increased markedly for GBF (from 53 to 80%) and also, but modestly, for GPACE (from 59 to 63%). In contrast, VBF and PACE did not show any differences, as most participants in both groups were able to lower their average breathing rate to the desired range during the initial training session. These results suggest that GBF can be as effective as VBF and PACE in helping participants lower their breathing rate, *if they are trained properly* to control their breathing in the initial training session.

#### Skill Transfer

**Figure 10:**
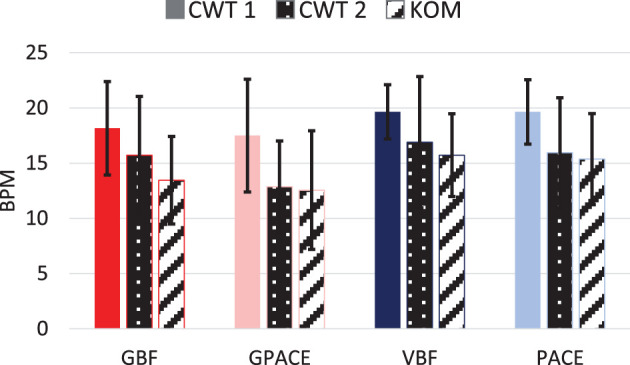
Average breathing rates during CWT 1, CWT 2, and KOM for all participants.

Next, we examined whether the treatments were able to reduce the breathing rates at post-test (CWT2 and KOM tasks) compared to pre-test (CWT1 task). For this purpose, one participant in the VBF group and one participant in the GPACE group were removed, as they did not perform any treatments sessions during the day. Results for the remaining participants are shown in Figure 10. Two-way ANOVA with treatment (4 conditions) and time (CWT1 vs. CWT2) as independent factors shows no significant effect for treatment and no interactions, but a significant effect for time [$F\left(3,64\right)=10.05,p=0.002,\;\eta_{time}^{2}=0.14$], which indicates that breathing rates during CTW2 (15.3) were lower than during CTW1 (18.73). Next, we performed two-way ANOVA with treatment (4 conditions) and time (CWT1 vs. KOM) as independent factors. As before, we observed no significant effect for treatment and no interaction, but a significant effect for time [$F\left(3,64\right)=21.38,\;p<0.001,\;\eta_{time}^{2}=0.25$], indicating lower breathing rates during KOM (14.27) than during CWT1 (18.73). These results combined indicate that there was skill transfer to both tasks at post-test, a more challenging version of the Color Word Test (shorter response time) and a previously unseen task (KOM).

#### Subjective Analysis

As in Experiment 1, participants were asked to fill a survey regarding their experience with the treatment. When participants were asked participants if they felt relaxed immediately after using their assigned intervention, 90% of the participants in the PACE group felt relaxed, compared to 64% for the GBF group, and 80% for VBF and GPACE. However, this result only tells part of the story, since the goal of the GBF intervention is to teach participants to lower their breathing rate when engaged in stressful tasks at a later time. Thus, we also asked participants if they were able to perform deep breathing after the respective breathing guide had been removed (pacing or biofeedback). In this case, only 30% of the participants in both the PACE and the GPACE groups answered that they were able to perform deep breathing after removing the pacing signal in the presence of a stressor, whereas 80% of participants in the GBF and VBF groups answered that they were able to perform deep breathing after removing biofeedback.

## Discussion

The objective of this study was to compare two types of breathing guides, biofeedback and pacing, to promote deep breathing. In addition, we also sought to determine how suitable the two breathing guides would be when integrated into a casual game. Integration with the game served two purposes. First, the game served as a minor stressor during deep breathing that was aimed at promoting skill transfer to a new task. Second, the game aimed to increase the enjoyability of the intervention, as a way to promote adherence to regular practice. We used a 2 × 2 design with game presence and type of breathing guide as independent factors, leading to four experimental treatments: biofeedback without the game (visual biofeedback; VBF), biofeedback with the game (game biofeedback; GBF), pacing without the game (pacing; PACE), and pacing with the game (game with pacing; GPACE).

In Experiment 1, conducted in a laboratory setting, we found that the four experimental interventions led to a pre-post treatment reduction in breathing rates while participants completed two stressor tasks (color word test, mental arithmetic), but that the differences were only significant for the GBF condition. We also found that adding a game component to the intervention made it more enjoyable.

Which guide is more suited for integration into a game: biofeedback or pacing? Participant feedback at the end of the experiment indicated that biofeedback integration in the game was perceived as helpful, whereas pacing integration into the game was perceived as distracting. In hindsight, this result is not surprising because the breathing exercise in GBF is deeply integrated into the game (i.e., it alters the gameplay), whereas the pacing signal in GPACE is only shallowly integrated (i.e., it has no effect on gameplay.) As a result, when practicing with GPACE, the player has to perform two disconnected tasks, which lead some participants in our study to choose between one or the other task, but not both.

In Experiment 2, we compared the four experimental treatments when performed in free-living conditions outside the laboratory. Namely, participants were trained to use the interventions and then asked to practice them whenever they saw fit during the day. Participant ratings in Experiment 1 had indicated that adding a game component to the breathing exercise led to higher enjoyment. But would higher enjoyment translate into higher usage statistics during the day? Results from Experiment 2 provide evidence of it: a *t*-test showed that the two game interventions were used more often than the non-game interventions, and a 2-way ANOVA showed a marginally significant effect for the inclusion of a game into the breathing exercise. A major finding in Experiment 2 was the need for proper training if participants are to perform the game-based interventions effectively in the wild. Namely, when we examined the percentage of treatment sessions where participants reached the effective breathing rate, we found a significant difference between the two non-game interventions, with success rates close to 85%, and the two game interventions, which rates around 50%. A likely explanation for this result is that the two non-game interventions are single tasks, where the user has to either follow a pacing signal (PACE) or monitor a numerical indicator displaying their breathing rate (VBF). As such, both tasks can be learned in a short period. In contrast, the two game-based interventions require multi-tasking, so performing them is likely to require more training. In the case of GPACE, the user must learn to synchronize their breathing rate with the pacing signal while they play the game. In the case of GBF, the user must be mindful of their breathing while playing the game to ensure it does not increase above threshold, and develop recovery strategies if it does. Fortunately, results from the experiment indicate that participants in the GBF group who were successful during training (i.e., in the lab) had similar success rates during treatment (i.e., in the wild) as those in the PACE and VBF condition, which indicates that the intervention can be trained in a relatively short period.

Our work is related to a study by Zafar et al. (52), who compared the skill transfer effectiveness of games with and without biofeedback, and a paced-breathing application. The authors used three custom-developed casual games and an off-the-shelf paced-breathing application to teach self-regulation skills. They observed that the biofeedback-game groups and the paced-breathing group obtained the same level of skill transfer. Our results, in contrast, show that only the GBF condition led to a significant reduction in breathing rates pre-post-test. This discrepancy between the two studies may be due to differences in the experimental protocol: in their study, participants practiced with the breathing application for 8 min, whereas participants in our laboratory study practiced for 30 min. Also related to our work, a recent study by Bockeler et al. (53) examined the effectiveness of paced breathing vs. a commercial biofeedback game based on heart rate variability (HRV). The authors found that HRV biofeedback led to a better vagal response (increase in low-frequency HRV) compared to paced breathing, and that HRV biofeedback was less stressful than being asked to follow a pacing signal. Since HRV generally increases with slow diaphragmatic breathing, this suggests that HRV may be an alternative to measuring breathing rate. One advantage of HRV is that it can be measured using wrist-based devices, whereas respiration rate generally requires wearing a chest strap, which is less comfortable to the user. However, HRV biofeedback is less intuitive than respiratory biofeedback, since respiration is under voluntary control. To address this point, in an earlier study (6), we compared three versions of a biofeedback game that were driven by either breathing rate, HRV, or electrodermal activity (EDA). We found that breathing-based game biofeedback was more effective in inducing relaxation during treatment than the HRV-based or EDA-based game biofeedback. In that earlier study, participants in the breathing-based group also showed greater retention of the relaxation skills (without biofeedback) during a subsequent stressor than participants in the HRV or EDA biofeedback groups.

Our study has several limitations that are worth noting. First, as noted in section Treatment effectiveness, the proportion of successful trials for participants in the two game-based groups was substantially lower than in the other two conditions. However, this difference disappeared when we only considered participants who had reached the effective breathing rate during the training session. This indicates that a single training session was insufficient for many participants. This issue could be addressed in future work by increasing the number of training sessions until participants can achieve proper control of their breathing rate. A second limitation of this study is the lack of an independent objective measure of relaxation, such as heart rate variability or electrodermal activity, to confirm that the interventions lead to physiological relaxation beyond a lowering of breathing rates. A potential concern is with the experimental protocol, since all participants performed a paced-breathing (PB) session. Thus, it is possible that this PB session could have affected the breathing rates at post-test. If that were the case, we would expect to see pre-post-test reductions in breathing rate for participants in the control condition, who also did the PB session. However, the results in Figure 5 and *post-hoc* analysis indicate that the breathing rate for participants in the control condition was similar during CWT1 and CWT2, which suggests that the initial PB session did not affect breathing rates at post-test. Likewise, it may be possible that training to play the base game (TRAIN in Figure 5) could have also influenced breathing rates at post-test. The results in Figure 5, however, indicate that the breathing rates during TRAIN were similar to those during CWT1, which suggests that the brief training session with the game did not alter breathing rates. Finally, the ambulatory study was relatively short (7 h on average), so it does not allow us to examine whether adherence to treatment reduces over time. These three limitations are being addressed in a forthcoming study, where participants will receive more extensive training while their electrodermal activity is monitored, and will be asked to perform the interventions for 3 days in free living conditions.

A primary objective of this study was to determine if a simple pacing signal would be as effective as game biofeedback. Results from Experiment 2 indicate that the choice between the two approaches depends on whether one seeks to induce relaxation during practice or promote skill transfer to a stressful situation. As noted in section Subjective analysis, 90% of participants in the PACE group reported feeling relaxed immediately after completing the intervention, whereas only 60% of participants in the GBF group reported the same. In contrast, only 30% of participants in the PACE group reported being able to perform deep breathing with a stressor once the breathing guide was removed, compared to 80% of the participants in the GBF group. Thus, these results suggest that, if the goal is to induce a feeling of relaxation in the short term, practicing with a pacing signal is more effective than practicing with game biofeedback. However, if the goal is to internalize deep breathing so it can be performed during a stressful task, practicing with game biofeedback is more effective than practicing with a pacing signal. According to this, both approaches have their time and place, and can be useful tools in the arsenal to combat the effects of chronic stress.

## Data Availability Statement

The datasets presented in this article are not readily available because this provision was not included in the consent form. Requests to access the datasets should be directed to rgutier@tamu.edu.

## Ethics Statement

The studies involving human participants were reviewed and approved by Texas A&M University Institutional Review Board. The patients/participants provided their written informed consent to participate in this study.

## Author Contributions

VG developed the mobile micro-interventions, conducted the user studies, and participated in the design of the experiments and data analysis. DS participated in the design of the experiments and data analysis. RG-O conceived the interventions, participated in the design of the experiments and data analysis, and prepared the manuscript for submission. All authors contributed to the article and approved the submitted version.

## Funding

This work was funded by the National Science Foundation under Award #1704636.

## Conflict of Interest

The authors declare that the research was conducted in the absence of any commercial or financial relationships that could be construed as a potential conflict of interest.

## Publisher's Note

All claims expressed in this article are solely those of the authors and do not necessarily represent those of their affiliated organizations, or those of the publisher, the editors and the reviewers. Any product that may be evaluated in this article, or claim that may be made by its manufacturer, is not guaranteed or endorsed by the publisher.

## Footnotes

^1^Other popular “endless” games include runner-type games such as Temple Run or Crossy Road.

^2^Instrumental conditioning is the process of rewarding or penalizing user based on their response to modify a target behavior. Negative reinforcement occurs when the target behavior is strengthened by removing an averse stimulus (46).

^3^Following our prior work (7), we also provide peripheral visual cues by displaying the breathing rate numerically on one of the corners of the screen, along with an arrow indicating whether the breathing rate is decreasing (green and downwards) or increasing (red and upwards). Further, a text prompt “*Please try and relax*” is shown at the bottom of the screen if the player's breathing rate is constantly increasing for more than 30 s. In that earlier work, we found that including these peripheral visual cues resulted in faster training of deep-breathing skills, but otherwise did not improve skill transfer.

^4^Institutional Review Board approval number: IRB2019-0218D.

^5^Results from Experiment 1 showed that the control condition was unable to reduce participants' breathing rates post-treatment. For this reason, the control condition was not considered for the ambulatory study.

^6^The relaxing video named “*Alaska's wild, Denali”* (49), which was shown to induce a neutral physiological response (50).

^7^The Bioharness sensor only provides an average breathing rate, which takes 45 s to converge and has a ±1 bpm accuracy (51). Further, the resonant breathing frequency is participant-dependent and can range from 5.5 to 6.5 bpm (28). For these above reasons, we established a 15% margin of 6 *bpm*±0.9 *bpm* as the effective breathing rate range.

^8^In Experiment 1 we had observed that, for all interventions, the average breathing rate during the six treatment sessions was very similar. This suggested that participants had learned to lower their breathing rate (and maintain it) within the first treatment session –see Figure 6.
